# Gender equity at last: a national study of medical students considering a career in rural medicine

**DOI:** 10.1186/s12909-020-02355-3

**Published:** 2020-11-16

**Authors:** Caleb Kim, Hanh Ngo, Denese Playford

**Affiliations:** Rural Clinical School of WA, School of Medicine, UWA, 35 Stirling Highway, Crawley, WA 6009 Australia

**Keywords:** Gender, Rural intention, Rural clinical school, Rural workforce

## Abstract

**Background:**

The rural medical workforce internationally suffers from a significant imbalance between male- and female- identifying practitioners. Not only do male doctors outnumber female doctors, but additionally female doctors work fewer hours than their male counterparts. This has health implications for rural communities. In response, In Australia, Rural Clinical Schools (RCSs) are a national training strategy to increase the number of graduates entering the rural medical workforce. It has been observed that RCSs attract a greater number of female students than male students. However, the future work intentions of male versus female RCS students is not known. This paper therefore asked whether male and female RCS students have equivalent intent for future rural practice.

**Methods:**

Participants were all students who attended RCSs from 2015 to 2017, who completed an exit survey that gathered data on demographic, experiential and intentional variables. Univariate analyses examined differences between the sexes. A multivariate model was constructed to determine the independent predictors for rural intention.

**Results:**

There were 2017 respondents across the 3 years, of whom 937 identified as male, and 1138 identified as female. In univariate analysis, female-identifying students had significantly higher rural intention than male-identifying students. There were no other sex-based differences in age, rural background, overall perception of support, and overall excellence of clinical education whilst in RCS.

However, in multivariate analysis, sex was not a significant predictor for rural work intention, whereas older age, rural background, and first preference for RCS were all predictive of increased rural intent, as expected from the literature. There were no differences between male and female students in their perceptions of the overall support and the clinical education provided by RCS.

**Conclusion:**

We conclude from this national study that sex is not an independent predictor for future rural work intention among RCS students. Considering the disproportionate number of female students entering RCS, this is reassuring for ultimately achieving rural workforce gender equity.

## Background

In addition to lower number of doctors per capita in the rural medical workforce, internationally the rural medical workforce also has fewer female than male practitioners [1–3]. This has been attributed to characteristics of rural medical work such as higher rates of on-call, fewer colleagues, and higher acuity presentations [4].

.Australia suffers from the same sex-imbalance in its rural medical workforce as elsewhere [5, 6], with fewer female-identifying general practitioners (GPs) compared to male-identifying GPs in regional and remote Australia [7]. This is a long-standing inequity [8] with even the most recent data from an Australian rural medical employment agency reporting that only 41% of the rural GP workforce identified as female [9]. The gender imbalance is further compounded by the fact that rural female GPs in Australia tend to work fewer hours compared to their male colleagues [10].

.The lack of female medical practitioners has important health implications for rural communities, as female doctors are generally preferred for female-specific healthcare [11], tend to practice a more patient-focused style of medicine, and attend to more psychosocial issues [5, 6] which is significant given a current rural mental health crisis, attributed to the vicissitudes of global warming [12].

.To address the general deficit in rural doctors, The Commonwealth Government of Australia has provided financial support to universities, specifying that 25% of all domestic medical students are to complete at least 1 year of their training at Rural Clinical Schools (RCSs) based in locations established as rural or remote according to an evolving classification system anchored to the Australia Bureau of Statistics [13]. A substantial body of evidence has demonstrated the effectiveness of RCSs at increasing the number of graduates going on to practice rurally, more so among those with rural intent or rural background [14, 15]. Additionally, there is some evidence to suggest that participation in RCSs encourages graduates to practice in more remote locations in contrast to regional areas [14, 16, 17].

.It is interesting to note that the RCS program is reported to recruit a greater number of female-identifying students compared to male-identifying students, more so than the recent increases in female to male proportions in medical school [18–21]. However, while a considerable volume of evidence shows that RCSs have a significant impact on graduates’ career paths, there is a dearth of evidence that specifically explores differences in students’ attitudes towards rural work that may underlie the existing sex difference, and thus it is unknown whether the more equitable balance of females to males in medical school is likely to carry downstream to the rural workforce.

Incidental findings suggest that differences based on sex do exist. For example, when considering location of work, female medical students valued opportunities for their children higher than their male counterparts, whereas male students valued their opportunities for their spouses higher than female students did [22]. Female RCS students had a greater preference for rural placements compared to male students [19] and displayed higher levels of cooperativeness and self-efficacy, both of which are positively associated with intention for rural work. However, studies conflict on whether a medical students’ sex is associated with intention for rural practice [21, 23–26] and actual future rural work [14, 27, 28] and no studies have specifically investigated the association between gender, intention for rural work and the factors contributing to this intention.

A comprehensive exploration into whether differences exist between male and female RCS students is warranted for two reasons. Firstly, these findings may establish whether there are fundamental differences in perceptions of rural work between male and female medical students that have implications for gender balance in the future rural workforce. Secondly, we will be able to assess whether the effect and experience of RCS are equitable across the sexes. Thus this national study of RCS students from 2015 to 2017 aimed to investigate the influence of sex on factors that contribute to rural career intent.

## Methods

### Design

This is a longitudinal case control study on survey data collected from all medical students in RCS who completed the Federation of Australian Medical Educators (FRAME) survey from 2015 to 2017, initially comparing female to male responses. The survey is conducted annually, and is administered by participating RCS’s to their students just before they complete their one or more RCS year/s. It collects information about students’ demographics, RCS experience, and intention for future rural work. Survey reports, as well as the actual surveys, are accessible via http://www.ausframe.org.

### Participants

To participate in the RCS program, 25% of Commonwealth supported medical students are selected by each RCS and moved by their university to one of the university’s rural catchment towns, some by choice and some by allocation, for a period of one or more years. Since the study population for this study was the entire RCS cohort from 2015 to 2017, a sample size calculation was not necessary.

### Demographic information

The FRAME survey collected demographic information on date of birth, sex, and rurality of students’ background. We note that, since the FRAME survey offered only a binary Male/Female choice, we can only discuss the outcomes as binary, with reference to the physical sexual characteristics of the student. We then go on to use the terms “male–identifying” / “female–identifying”, “sex”, and “gender” interchangeably, understanding that the reality of gender is far from binary and in so doing, we also acknowledge that binary categories in themselves may be offensive to some, but that we refer to them as a simple reflection of the data as we have it. In this study, participants’ age was dichotomized into < 25 and ≥ 25 years old, and rurality of background as either rural or urban.

### Dependent variable: intention for rural practice

Intention to practice in a rural location was measured by asking participants to rank their preferred location of practice upon completion of training using the Remoteness Areas classificaon (capital or major city, inner regional city or large town in Australia (25,000 – 100,000), smaller town in Australia – outer regional (10,000 – 24,999), small rural or remote community in Australia (< 10,000), and very remote centre/area [13]. In this study, intention was designated as the outcome variable and was dichotomised into rural (first preference location other than ‘capital or major city’) and urban intention (first preference as ‘capital or major city’). Students’ preferred specialties on both commencement and completion of their RCS year were dichotomised into ‘GP’ or ‘specialist’.

### Experiential factors

Questions that measured students’ perceptions of their RCS experience were collected by asking students to rate their agreement with statements provided on the survey with a 5-point Likert scale. Responses to Likert scale questions were dichotomised into strongly agree/somewhat agree (coded as ‘agree’) and neutral/disagree/strongly disagree (coded as ‘not agree’). If the distribution was skewed such that there was more than 80% of the data in one category, the variable was categorised into three levels.

### Statistical analysis

De-identified survey data was provided to the research group in the form of electronic spreadsheets and were imported into statistical software. Analyses were conducted using IBM SPSS Statistics version 25 (https://www.ibm.com/products/spss-statistics). As the FRAME survey is administrated by a consortium to which new questions can be submitted, unique survey questions were found across the different FRAME surveys. For our purpose, therefore, only questions that were common across all 3 years were included in the analysis.

The χ^2^ test was used to determine differences in demographics, rural intention, and experience between male and female identifying students. Demographic, intentional, and experiential variables that predicted rural intention were first analysed using the χ^2^ test and significant predictors were added to the multivariate model. Backwards stepwise binary logistic regression was used to eliminate insignificant variables. Variables’ contribution to intention was analysed for interaction with sex.

Cases with missing data were excluded from the analyses on a variable by variable basis.

### Ethics approval

This study was approved by the University of Western Australia Human Research Ethics Committee (RA/4/1/4579).

## Results

### Participants

There were 2017 respondents across the 3 years, with number of respondents per annum increasing slightly (Table 1), and 56.4% of respondents identifying as female. The response rate for 2015 was 641 of 788 enrolled RCS students across Australia (81.3%)ok; for 2016 was 630 of 785 RCS students (80%), and for 2017 was 721 of 841 RCS students (86%).

**Table 1:** Demographic data for students who completed the FRAME survey from 2015 to 2017 inclusive

Variab	n (% of total)
Year	
2015	644 (31.9%)
2016	677 (33.6%)
2017	686 (34.5%)
Total	2017 (100%)
Age	
Less than 25	1033 (51.2%)
25 and older	937 (46.5%)
Missing	47 (2.3%)
Gender	
Female	1138 (56.4%)
Male	851 (42.2%)
Missing	28 (1.4%)
Considers self to be from rural background	
Yes	870 (43.1%)
No	1116 (55.3)
Missing	31 (1.5%)
Intended location of practice upon completion of training	
Rural	736 (36.5%)
Urban	1262 (62.6%)
Missing	19 (0.9%)

### Sex differences

Male and female students did not differ in terms of demographic variables, with age (*p* = 0.257), rural background (*p* = 0.966) and area lived longest (*p* = 0.654) being statistically indistinguishable (Table 2). However, male and female students consistently differed in intention for rural practice. In univariate analyses female sex was positively associated with 1st preference for RCS (*p* = 0.002), rural intention (*p* < 0.001), intent for general practice (*p* < 0.001), and agreement with the statements that RCS increased interest for rural (*p* < 0.001) and general practice (*p* < 0.001). Female respondents also had stronger positive feelings about working in a rural setting (*p* < 0.001) and agreed more with the statement that they saw people like themselves working in a rural setting (*p* < 0.007). The association between sex and increased remote interest did not reach statistical significance (*p* = 0.077).

**Table 2:** Univariate associations, comparing female with male RCS students

Variable	*N* = male respondents (%)	*N* = female respondents (%)	*P*-value (χ2 test)
Age			0.257
Less than 25 years	425 (50.9%)	596 (53.6%)	
25 years and older	410 (49.1%)	516 (46.4%)	
Rural background			0.966
Yes	370 (44.1%)	493 (43.9%)	
No	469 (55.9%)	630 (56.1%)	
Which area have you lived longest?			0.654
Urban	488 (57.7%)	641 (56.7%)	
Rural	358 (42.3%)	490 (43.3%)	
Intention for work upon completing training			< 0.001
Urban	353 (41.6%)	375 (33.3%)	
Rural	495 (58.4%)	750 (66.7%)	
Preference for RCS			0.002
First preference	545 (64.4%)	803 (71.1%)	
Not first preference	301 (35.6%)	326 (28.9%)	
Preferred specialty upon RCS entry			< 0.001
GP	189 (22.3%)	370 (32.8%)	
Specialist	659 (77.7%)	758 (67.2%)	
Preferred specialty upon RCS exit			< 0.001
GP	185 (21.8%)	355 (31.4%)	
Specialist	663 (78.2%)	774 (68.6%)	
RCS has increased GP interest			< 0.001
Strongly agree/somewhat agree	480 (56.6%)	728 (64.5%)	
Not agree	368 (43.4%)	401 (35.5%)	
RCS has increased rural interest			< 0.001
Strongly agree	326 (38.4%)	539 (47.7%)	
Somewhat agree	358 (42.2%)	455 (40.3%)	
Not agree	165 (19.4%)	136 (12%)	
RCS has increased remote interest			0.690
Strongly agree/somewhat agree	344 (40.6%)	505 (44.7%)	
Not agree	504 (59.4%)	626 (55.3%)	
I would recommend RCS to others			0.026
Strongly agree	618 (73.5%)	876 (78.6%)	
Somewhat agree	159 (18.9%)	177 (15.9%)	
Not agree	64 (7.6%)	62 (5.6%)	
Clinical supervisors provided adequate clinical responsibilities			0.037
Strongly agree	347 (40.8%)	526 (46.5%)	
Somewhat agree	394 (46.4%)	479 (42.4%)	
Not agree	109 (12.8%)	125 (11.1%)	
Clinical supervisors provided appropriate supervision			0.003
Strongly agree	352 (41.5%)	554 (49%)	
Somewhat agree	397 (46.8%)	474 (41.9%)	
Not agree	99 (11.7%)	103 (9.1%)	
Overall perception of support whilst on RCS			0.055
Strongly agree	414 (48.7%)	601 (53.1%)	
Somewhat agree	322 (37.9%)	370 (32.7%)	
Not agree	114 (13.4%)	161 (14.2%)	
Overall positive impact of RCS on wellbeing			0.072
Strongly agree	398 (46.9%)	587 (52%)	
Somewhat agree	276 (32.5%)	339 (30%)	
Not agree	175 (20.6%)	203 (18%)	
I felt socially isolated during my RCS placement			0.627
Strongly agree/somewhat agree	258 (30.4%)	356 (31.5%)	
Not agree	592 (69.6%)	775 (68.5%)	
My clinical supervisors treated me with respect			0.056
Strongly agree	466 (54.9%)	681 (60.2%)	
Somewhat agree	320 (37.7%)	371 (32.8%)	
Not agree	63 (7.4%)	80 (7.1%)	
Overall, my clinical school provided an excellent clinical education			0.092
Strongly agree	464 (54.7%)	653 (57.8%)	
Somewhat agree	290 (34.2%)	382 (33.8%)	
Not agree	95 (11.2%)	95 (8.4%)	
Rural practice is too hard			0.029
Strongly agree/somewhat agree/neutral	155 (18.3%)	167 (14.8%)	
Somewhat disagree	495 (58.5%)	649 (57.7%)	
Strongly disagree	196 (23.2%)	309 (27.5%)	
I get a sinking (anxious) feeling when I think of working in a rural setting			0.097
Strongly agree/somewhat agree/neutral	186 (22%)	208 (18.5%)	
Somewhat disagree	371 (43.8%)	491 (43.6%)	
Strongly disagree	290 (34.2%)	426 (37.9%)	
I get a strong positive feeling when thinking about working in a rural setting			< 0.001
Strongly agree/somewhat agree	578 (68.2%)	850 (75.5%)	
Not agree	269 (31.8%)	276 (24.5%)	
I see people like me taking up rural practice			< 0.006
Strongly agree/somewhat agree	534 (63.2%)	778 (69.1%)	
Not agree	311 (36.8%)	348 (30.9%)	

These differences were not based on differences in experience, as there was no significant gender difference among students when asked to rate the overall support experienced on RCS (*p* = 0.055), overall excellence of clinical education (*p* = 0.092), overall positive impact of RCS on their wellbeing (*p* = 0.072), or experience of social isolation (*p* = 0.627).

### Predictors of rural intent

In multivariate analysis, the association between female-identification and intention for rural work did not retain significance, and thus sex was not included in the final multivariate model. Other demographic variables that were significantly related to intention (Table 3) included older age (OR, 1.52; 95% CI, 1.21–1.92) and rural area lived longest (OR, 3.81; 95% CI, 2.95–4.93). Variables that were also associated with rural work intention were pre- (OR, 1.52; 95% CI 1.10–2.11) and post- (OR, 1.48; 95% CI, 1.03–2.14) RCS intention for pursuing a career in general practice, putting RCS as first preference (OR, 1.49; 95% CI, 1.16–1.9), having strong positive feelings about working in a rural setting (OR, 1.8; 95% CI: 1.32–2.47), and disagreeing with the statement that they felt anxious about rural work (OR, 1.61; 95% CI, 1.16–2.25). Students who expressed strong agreement that RCS clinical supervisors gave constructive feedback were less likely to express rural intent (OR 0.53 95% CI 0.34–0.81). Conversely, students who strongly agreed that their supervisors were excellent role models were more likely to express rural intent (OR, 1.89; 95% CI 1.12–2.93).

**Table 3:** Final multivariate model for factors associated with rural intention

Factor	OR (95% CI)
Age	
25 years old or greater	1.52 (1.21–1.92) ***
Less than 25 years old^a^	
Area lived longest	
Rural	3.81 (2.95–4.93)***
Urban^a^	
Preferred specialty before commencing RCS	
GP	1.52 (1.1–2.11)**
Specialist^a^	
Preferred specialty upon completing RCS	
GP	1.48 (1.03–2.14)*
Specialist^a^	
RCS has increased interest in pursuing a career in regional or rural Australia	
Strongly Agree	3.16 (2.14–4.66)***
Agree	1.51 (1.06–2.15)*
Not agree^a^	
RCS clinical supervisors gave me constructive feedback	
Strongly Agree	0.53 (0.34–0.81)*
Agree	0.93 (0.65–1.33)
Not agree^a^	
RCS clinical supervisors were excellent role models	
Strongly Agree	1.89 (1.22–2.93)***
Agree	1.41 (0.96–2.08)
Not agree^a^	
Preference for RCS	
First preference	1.49 (1.16–1.9)***
Not first preference^a^	
I have necessary skills to practice in a rural setting	
Strongly agree/somewhat agree	0.59 (0.44–0.77)***
Not agree^a^	
I get an anxious feeling when I think of working in a rural setting	
Strongly disagree	1.61 (1.16–2.25)**
Disagree	2.4 (1.64–3.52)***
Not disagree^a^	
I have a strong positive feeling when I think of working in a rural setting	
Strongly agree/somewhat agree	
Not agree^a^	1.8 (1.32–2.47)***
People tell me I should work in a rural setting	
Strongly agree/somewhat agree	1.38 (1.06–1.78)*
Not agree^a^	
I see people like me taking up rural clinical practice	
Strongly agree/somewhat agree	1.35 (1.03–1.77)*
Not agree^a^	
Membership in rural health club	
Yes	1.66 (1.31–2.1)***
No^a^	
Participation in the John Flynn Scholarship Program	
Yes	1.71 (1.14–2.57)**
No^a^	

*p < 0.05, **p < 0.01, ***p < 0.001

^a^ Denotes reference group, OR = 1.0

## Discussion

This is the first national study of RCS students that has specifically evaluated the association between sex and intention for future rural practice. In univariate analysis, female sex was significantly associated with rural intent. Females also had a higher preference for GP training, which is reported to be associated with future rural practice [29]. At this level of analysis, female students were also more likely than males to identify their RCS experience as a factor that increased rural interest. However, when all factors were taken into account concurrently in the multivariate model, sex did not retain significance as a predictor of rural intent. This finding corroborates the incidental reports of numerous smaller, single-centre studies that examined rural intention [26, 28] and work [14, 17, 20, 27, 28, 30] but did not principally investigate the effect of gender.

The validity of our multivariate model is further endorsed by our confirmation of factors already known to be associated with rural intention: older age [14], rural background [15], RCS as first preference [19], GP intention on entry [21] and increased interest in pursuing a rural career [21]) were all significant to rural intent in our analyses, suggesting that the lack of association we identified between sex and rural intention is a real and strong finding from this national-level analysis.

Another study using results from the FRAME survey has indirectly supported the validity of our model. Isaac et al used six questions of the FRAME study that measured self-efficacy to construct a self-efficacy variable, which was demonstrated to be significantly associated with rural intent [21]. In our model using more recent data, four of these self-efficacy variables were similarly associated with rural inclination. It is reassuring that in our cohort, students who had strong positive feelings towards rural work, concurrently disagreed that they experienced anxious feelings towards rural work. These positive students additionally said they identified with others who took up rural practice. They also reported that they received affirmation from others to pursue rural practice. They were more likely than their other peers to indicate preference for future rural work. For these students, their constellation of character traits may respond to the challenges surrounding rural work as a positive incentive, spurring them on to a rural career. A similar pattern of increased resilience as a character trait has been identified among the subgroup of general practice registrars who went on to rural work [31].

.When translated to real-world outcomes, the results from this study are encouraging as they stand in contrast with the historically diminutive numbers of female medical practitioners in the rural workforce [32]. When added to the recent demographic shift in medical school towards a female majority [18–21, 33], the fact that both female and male students are indistinguishably interested in rural work is a reassuring finding, suggesting that sex-based equity is achievable. It must be noted that female practitioners tend to work fewer hours than their male counterparts [9], and thus greater numbers are needed to actually achieve equity. Gender equity has positive implications for health outcomes, especially for female-specific medicine [11], and mental health issues, given that female doctors’ consults are generally longer in duration [34] and more holistic in their approach [35] with a greater emphasis on psychosocial issues [36], possibly identifying issues that shorter consults would not pick up.

Another reassuring finding is the lack of a significant difference between male and female students in their perception of overall support provided by RCS, positive impact of RCS on their wellbeing, and quality of clinical education received whilst on RCS. This demonstrates that from a pedagogical and a support perspective, the RCS experience appears to be equitable across the sexes. This stands in contrast to the findings of a qualitative study investigating medical student perceptions of rural work two decades ago, where female students reported that they felt intimidated about the difficulty of rural practice and the male-dominated culture [37].

.An area for further investigation would be to determine the reason for the shift towards gender equity. Walters et al have suggested [19] that the high number of female clinical academics in RCSs, as demonstrated by Playford et al. [30], may serve as positive role models for rural work to their female students. The authors speculate the shift may also be due in part to the downstream effects of the gradual feminisation of medicine [38], evidenced in the shift towards more females in medical school [18–21] and general practice [39].

.The present study is limited by the fact that we only looked at RCS students, without a non-RCS control. Thus, these findings may not be representative of the medical school cohort as a whole, especially given the intentional differences between the two student groups [15]. Indeed, a relative minority of urban-origin graduates are found in the rural workforce [15]. However, it must be noted that RCS participation is a key factor associated with future rural work, and we can thus validly use RCS data as we have, to infer downstream workforce effects [14]. Another limitation of the study is that intention has been used as a proxy for future medical work, instead of using actual work location. However, intention has been demonstrated as a predictor for future rural work [40, 41], and graduate tracking studies for individual universities have also shown gender equity in rural recruitment from RCS. Thus, our data suggest that a pan-Australian, longitudinal investigation of both urban and RCS students would be likely to arrive at similar conclusions.

The role of RCSs has been consistently recognised as a substantial contributor to increasing the number of doctors in the rural workforce. By establishing that there is no significant difference in rural intent between male and female students, our study has also importantly demonstrated that RCSs will also contribute to overcoming existing sex-based inequity in the rural workforce. Reassuringly, this study also demonstrates that RCSs are perceived as being equitable in their provision of learning and support to both male and female students.

## Data Availability

The datasets used during the current study are available from the corresponding author on reasonable request.

## Abbreviations

FRAME: Federation of Australian Medical Educators
GP: General practitioner
RCS: Rural Clinical School
